# Ascorbyl palmitate synthesis in an organic solvent system using a Celite-immobilized commercial lipase (Lipolase 100L)

**DOI:** 10.1007/s13205-016-0486-7

**Published:** 2016-08-27

**Authors:** Shivika Sharma, Kriti Kanwar, Shamsher S. Kanwar

**Affiliations:** Department of Biotechnology, Himachal Pradesh University, Summer Hill, Shimla, 171 005 India

**Keywords:** Celite, Lipase, Glutaraldehyde, Ascorbyl palmitate synthesis, Molecular sieves

## Abstract

Ascorbyl palmitate was synthesized using a Celite-immobilized commercial lipase (Lipolase 100L) in dimethylsulfoxide (DMSO) as an organic solvent system. Lipase immobilized by surface adsorption onto Celite 545 matrix and subsequently exposed to 1 % glutaraldehyde showed 75 % binding of protein. The Celite-bound lipase was optimally active at 75 °C and pH 8.5 under shaking and showed maximum hydrolytic activity toward *p*-NPP as a substrate. The bound lipase was found to be stimulated only in the presence of Al^3+^ and EDTA. All surfactants (Tween-20, Tween-80 and Triton X-100) had an inhibitory effect on lipase activity. The optimization of various reaction conditions of ascorbyl palmitate was achieved considering one factor at a time. The esterification of ascorbic acid and palmitic acid was carried out with 1 M ascorbic acid and 2.5 M palmitic acid in DMSO at 75 °C for 18 h under shaking (120 rpm). Molecular sieves had an important effect on the ester synthesis resulting in an enhanced yield. The by-product (H_2_O) produced in the reaction was scavenged by the molecular sieves (20 mg/ml) added in the reaction mixture which enhanced the ester yield to 80 %. The characterization of synthesized ester was done through FTIR spectroscopy.

## Introduction

Lipases bring about a range of bioconversion reactions such as hydrolysis, inter-esterification, esterification, alcoholysis, acidolysis and aminolysis (Rajesh and Reddy 2013). Lipases find potential applications in bioprocesses largely due to their availability and stability in organic as well as in aqueous media (Sharma and Kanwar 2014). The use of lipase in immobilized form ensures that the enzyme remains stable in the organic medium, facilitating its subsequent recovery (Padilha et al. 2013). Lipase-catalyzed production of flavor esters by transesterification reactions is influenced by a number of transesterification variables, such as molarity of alcohol, reaction time, temperature and amount of immobilized enzyme (Garlapati and Banerjee 2013). There are many advantages of using enzyme in low water/organic solvent media such as better solubility of substrate and products, simple removal of solvent (most of the organic solvents have lower boiling point than water), reduction in water-dependent side reactions, easy removal of enzyme after reaction since it is not dissolved in organic solvents, better thermal stability of enzyme at high temperature, elimination of microbial contamination, absence of undesirable side reactions and an increased thermal stability of the enzyme in harsh conditions. The stability of biocatalyst in an organic solvent such as DMSO might be attributed to the fact that a thin layer of molecules remain tightly bound to the enzyme acting as a protective sheath along the enzyme hydrophilic surface, allowing retention of native confirmation. Also, the stimulation effect of the solvent on the enzyme modifies the oil–water interface to make enzymatic action easier without causing protein denaturation. Enhancement in enzymatic activity in the presence of organic solvents could be due to some alterations in the catalytic hydrophobic pocket of the enzyme (lipase) containing serine, aspartic acid and histidine groups. Lipases are the most sought enzymes from the viewpoint of solvent stability and possibly because of their esterification and transesterification reactions which are favored in the non-aqueous media.

**Fig. 1:**
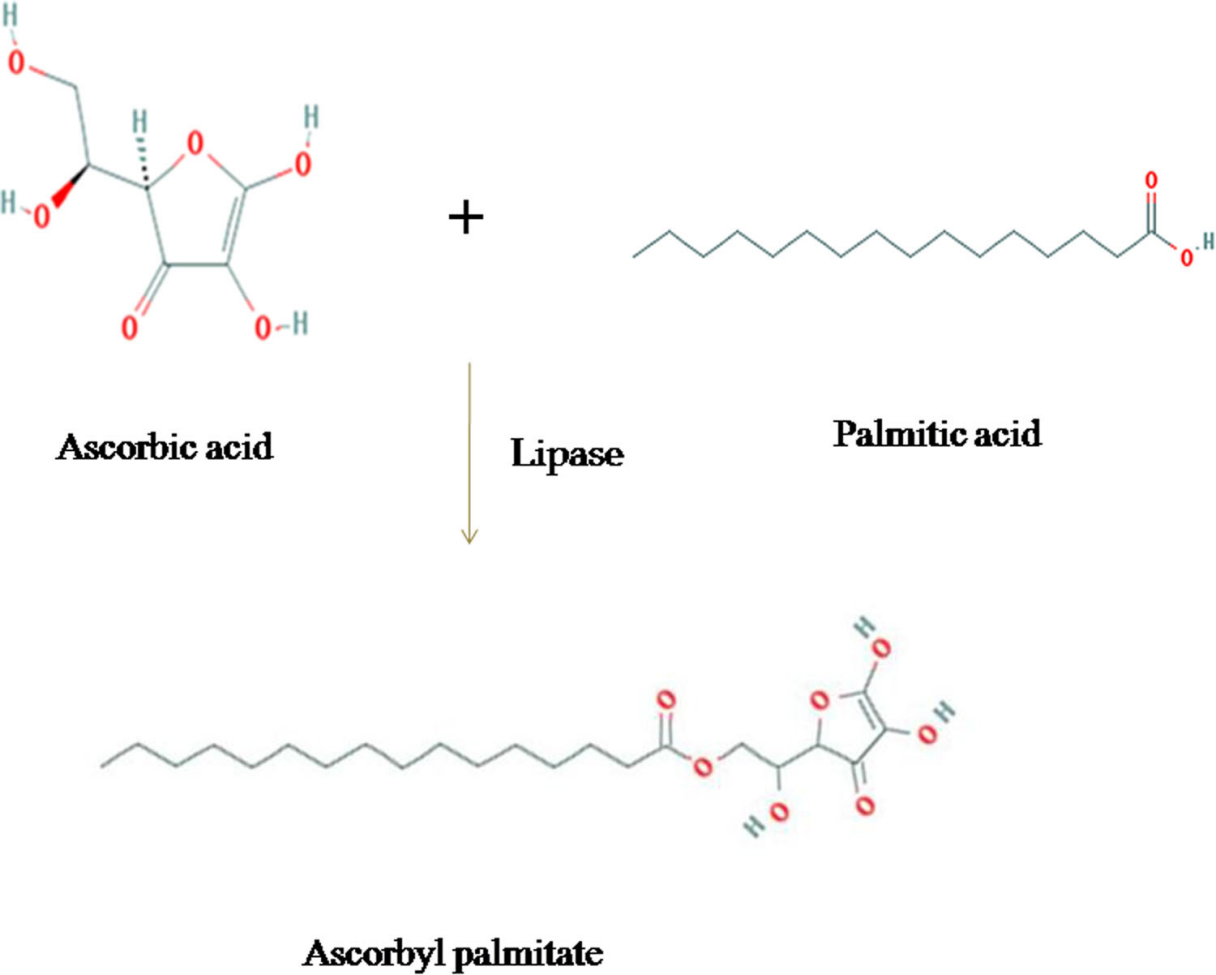
Schematic steps for the formation of ascorbyl palmitate using immobilized lipase

Ascorbyl esters are emerging food, cosmetic and pharmaceutical additives, which can be prepared in an eco-friendly way by using lipases as catalysts. Because they are amphiphilic molecules, which possess high free radical scavenging capacity, they can be applied as liposoluble antioxidants as well as emulsifiers and biosurfactants (Stojanovic et al. 2015). l-Ascorbic acid (vitamin C) is a natural antioxidant, but is highly polar and does not dissolve in fats and oil. The esterification process of converting ascorbic acid to its acid esters has been regarded as an effective solution for overcoming such problems. Furthermore, the esterified ascorbic acid products also have the antioxidant and surfactant functions with its potential application in high fat and food industries (Sun et al. 2013). The fatty acid and their esters obtained from oil and fats are used as acylating agent for the synthesis of hydrophobic vitamin C fatty acid ester (Karmee 2011). l-Ascorbyl palmitate is an amphipathic molecule that has both a hydrophilic (polar) head and a hydrophobic (a polar) tail and hence it is both lipohilic and water soluble (Fig. 1).

Ascorbyl palmitate is preferred over ascorbic acid as an ingredient in food due to its lipophilic nature (Coppen 1999). It also acts as an anticarcinogenic compound and can strongly inhibit the DNA synthesis in cancer cells (Kageyama et al. 1999). Ascorbyl palmitate is also used as an antioxidant and preservative in food, vitamins, drugs and cosmetics (Wawire et al. 2011). It is also used to control the oxidation of lipids such as PUFA (Jacobsen 2010). Ascorbyl palmitate also acts synergistically with vitamin E. In most prevalent manufacturing processes, ascorbic acid is esterified with sulfuric acid and the product of the reaction is esterified with palmitic acid (Pokorny et al. 2001).

Chemical synthesis has some drawbacks, such as the low or non-selectivity for the site of esterification, the requirement for protection and de-protection steps and product purification difficulties (Liang et al. 2012). However, vitamin C ester undergoes oxidation, degradation and rearrangement under the chemo-catalyzed reaction (Karmee 2011). Owing to a number of important applications of ascorbic acid or its derivatives, the aim of the present work was to synthesize ascorbyl palmitate in an organic medium using Celite-bound immobilized commercial lipase (Lipolase 100L).

## Materials and methods

### Chemicals and reagents

ZnCl_2_, MnCl_2_, MgCl_2_, CoCl_2_, HgCl_2_, AlCl_3_, CaCl_2_, NH_4_Cl_2_, *p*-nitrophenyl palmitate (*p*-NPP), *p*-nitrophenyl formate (*p*-NPF), *p*-nitrophenyl caprylate (*p*-NPC), *p*-nitrophenyl acetate (*p*-NPA), *p*-nitrophenyl benzoate (*p*-NPB), *p*-nitrophenyl laurate (*p*-NPL), *iso*-propanol, Tween-20, Tween-80, Triton X-100, EDTA, sodium citrate, Tris buffer (HIMEDIA Laboratory Ltd., Mumbai, India), palmitic acid (Acros Ltd, New Jersy, USA), Celite 545, DMSO and ascorbic acid (S.D. Fine-Chem Ltd., Hyderabad, India) were obtained. All these chemicals/reagents were of analytic grade and used as received.

### Commercial lipase (Lipolase 100L)

The commercial and purified lipase (Lipolase 100L) was manufactured by Novozyme A/S Denmark (MW 31,700 Da) and obtained from the Department of Biotechnology, Himachal Pradesh University, Summer Hill, Shimla (India), for further use.

### Assay of lipase activity

The hydrolytic activity of the lipase was assayed by a colorimetric method using *p*-NPP. The reaction mixture contained 80 μl of *p*-NPP (10 mM, *p*-NPP prepared in *iso*-propanol) stock solution and an appropriate amount of matrix-bound biocatalyst or free lipase. The final volume of this reaction mixture was made to 3 ml with 0.02 M Tris buffer, pH 8.5 (for free and immobilized lipase) with gum acacia (0.1 % w/v). The test tubes were incubated for 10 min at 45 °C under continuous shaking in a water-bath shaker. The absorbance of *p*-nitrophenol released was measured at 410 nm (Shimazdu UV/visible spectrophotometer, Japan). The enzyme activity was defined as µmole(s) of *p*-nitrophenol released per min by 1 ml of enzyme or 1 g of Celite-immobilized enzyme (weight of the matrix included) under standard assay conditions.

### Immobilization of lipase (Lipolase 100L) on Celite 545 matrix

Celite 545 (2 g) was incubated with 10 ml 0.02 M Tris HCl buffer of pH 8.5 for 24 h in a vial at 37 °C in a water bath. The enzyme (4 ml, 12.00 U/ml) was added to the matrix and incubated at 37 °C. Each of the matrices was then given five washings with Tris–HCl buffer (0.02 M), pH 8.5, to get rid of unbound enzyme. The matrix was then activated by 1 % cross-linking agent glutaraldehyde for 1 h at 37 °C in a water bath. Each activated matrix was further given five washings with Tris–HCl buffer (0.02 M), pH 8.5, to get rid of unbound activating agent. The weight of enzyme-incubated matrices was recorded and the activity was assayed using 5 mg of immobilized matrix using the standard protocol. The immobilized protein in the Celite matrix was determined by subtracting the unbound protein in the supernatant from the total protein used for immobilization as well as increased/decreased total activity was calculated by adding total activity of supernatant and matrix in comparison to total enzyme units in 4 ml of purified lipase incubated earlier.

#### Swelling capacity

The swelling capacity (Sw) of the selected Celite matrix in distilled water was found as follows;

$${\text{Sw}} = {\text{W2}} - {\text{W1}}/{\text{W1}}$$

W1: weight of dry matrix in g; W2: weight of wet matrix in g (i.e., net weight of matrix after suspending it in excess volume of water for 1 h at 75 °C).

#### Celite matrix

Immobilization by adsorption using inorganic matrices like Celite-545 has been most widely used for immobilization of many enzymes. Diatomaceous earth (Celite) is a naturally occurring, soft, chalk-like sedimentary rock that is easily crumbled into a fine white to off-white powder. This powder has an abrasive feel, similar to pumice powder and is very light, due to its high porosity. The use of a porous support material is desirable/recommended for immobilization of lipase, so that suitable amounts of lipase can be spread on a surface area without conformational changes.

### Optimization of hydrolytic properties of immobilized lipase

The effect of pH, temperature, specificity toward the hydrolysis of *p*-nitrophenyl esters with varying C-chain length, salt ions, chelating agents and various detergents was studied using Celite-bound lipase.

#### Effect of pH of reaction buffer on the activity of immobilized lipase

The activity of the Celite-immobilized lipase (5 mg) and free lipase (5 µl) was assayed separately by incubating the reaction cocktail at different pH ranges, viz. 6.0, 6.5, 7.0, 7.5, 8.0, 8.5, 9.0, 9.5 and 10.0 in a water-bath incubator under shaking (120 rpm) for 10 min at 75 °C and the lipase activity was measured thereof.

#### Effect of temperature on the activity of immobilized lipase

To study the effect of reaction temperature on the lipase activity, the Celite-immobilized lipase (5 mg) and free lipase (5 µl) were incubated in the reaction buffer at selected temperatures (50–80 °C) under shaking (120 rpm) for 10 min. The lipase activity at each of the selected temperatures was determined and compared.

#### Effect of *p*-nitrophenyl esters of varying C-chain length (substrate) on the activity of immobilized lipase

Substrate specificity of the lipase was investigated using stock(s) of *p*-nitrophenyl fatty acid esters of varying chain length (*p*-NPA, *p*-NPB, *p*-NPC, *p*-NPF, *p*-NPL and *p*-NPP) prepared in *iso*-propanol (10 mM) in the reaction cocktail and the lipase activity was assayed under standard conditions. The Celite-immobilized lipase was reacted with each of these substrates prepared in 0.05 M Tris buffer (pH 8.5) for 10 min at 75 °C under shaking (120 rpm). Thereafter, assay for the lipase activity was done.

#### Effect of salt ions on the activity of immobilized lipase

To evaluate the effect of various salt ions on Celite-immobilized lipase, an attempt was made to study the effect of a few of the selected salt ions, viz. Zn^2+^, Mn^2+^, Mg^2+^, NH_4_^+^, Co^2+^, Hg^2+^, Ca^2+^ and Al^3+^, on lipase activity. Each of the metal ions was separately included in the reaction mixture at a final concentration of 1 mM. The lipase activity was assayed under standard assay conditions at 75 °C (in Tris buffer 0.02 M) and pH 8.5 and the lipase activity was measured thereof.

#### Effect of chelating agents on the activity of immobilized lipase

The effect of chelating agents (EDTA and sodium citrate) on the activity of the Celite-immobilized lipase (5 mg) was studied by pre-incubating the Celite-bound lipase with the chelating agents at a final concentration of 1, 3 and 5 mM (in Tris buffer 0.02 M, 8.5 pH) for 10 min at 75 °C. The lipase activity was assayed under standard assay conditions at 75 °C and pH 8.5. The residual lipase activity was determined in each case and expressed as relative activity with respect to the control (without chelating agents).

#### Effect of detergents on the activity of immobilized lipase

The effect of each of the selected detergents (Tween-20, Tween-80 and Triton X-100) was also studied by including the detergents in the reaction mixture at a final concentration of 1, 5, 10 and 20 % (v/v) (in Tris buffer 0.02 M and pH 8.5). The reaction cocktail treated with lipase was incubated at 75 °C for 10 min and the lipase activity was measured thereof.

#### Reusability of Celite-immobilized lipase

The immobilized lipase (5 mg) was used repetitively up to the eighth cycle of hydrolysis at 75 °C under shaking. After the first cycle of reaction, the biocatalyst was recovered (by centrifuging and decanting the reaction mixture) and this biocatalyst was used to catalyze the fresh hydrolytic reaction.

### Ascorbyl palmitate synthesis using immobilized lipase

The efficacy of the Celite-bound lipase to perform catalysis in solvent medium was tested by performing esterification reactions of ascorbic acid and palmitic acid in DMSO (2 ml) at optimal temperature (75 °C) in a chemical reactor under shaking (120 rpm).

### Optimization of reaction conditions for synthesis of ascorbyl palmitate by immobilized lipase

Ascorbyl palmitate was synthesized using 1 M ascorbic acid, 2.5 M palmitic acid and Celite-bound lipase taken in a glass vial (20 ml capacity). The reaction was performed at 75 °C for 10 h in a chemical reactor. The effect(s) of incubation time, reaction temperature, relative molar concentration of reactants, biocatalyst concentration and concentration of molecular sieves on the rate of synthesis of ascorbyl palmitate was separately evaluated. The ascorbyl palmitate was separated on the basis of their solubility in hot water using a separating funnel. The ascorbic acid was soluble in hot water, while the formed ester (ascorbyl palmitate formed in different test tubes) was insoluble in hot water and separated out using a separating funnel. The amount of ester synthesized was determined and represented as % yield. The synthetic reactions in water-free medium were performed in triplicate and mean values were presented.

#### Effect of relative molar concentration of reactants on the synthesis of ascorbyl palmitate

It was studied by keeping the concentration of one of the reactants (ascorbic acid) constant at 1 M and varying the concentration of the other reactant (palmitic acid; 1, 2.5, 5 M) in an organic reaction mixture. The esterification was carried out at 75 °C for incubation time of 10 h under continuous shaking using Celite-bound lipase.

#### Effect of reaction temperature on the synthesis of ascorbyl palmitate

The reaction mixture (2 ml) contained 10 mg enzyme, 1 M ascorbic acid and 2.5 M palmitic acid in DMSO. The reaction mixture was incubated at 55, 65, 75 and 85 °C in the incubator shaker at 120 rpm for 10 h. The sample were extracted and weighed for the detection of ascorbyl palmitate.

#### Effect of incubation time for the synthesis of ascorbyl palmitate

The reaction mixture comprised Celite-immobilized lipase, 1 M ascorbic acid and 2.5 M palmitic acid taken in screw-capped Teflon-coated glass vials (5 ml capacity) in an organic system comprising DMSO, respectively. The glass vials were incubated at 75 °C in a chemical reactor at periodic (6 h) intervals, viz. 0, 6, 12, 18 and 24 h, under continuous shaking (120 rpm) for the synthesis of ascorbyl palmitic acid.

#### Effect of biocatalyst concentration on the synthesis of ascorbyl palmitate

The synthesis of ascorbyl palmitate was studied by taking different amounts of immobilized lipase (5, 10, 15, 20, 25 and 30 mg/ml) in a reaction mixture (2 ml) containing 1 M ascorbic acid and 2.5 M palmitic acid in an organic system at 75 °C. The reaction was carried out for 18 h and the sample was extracted and weighed for detection of ascorbyl palmitate.

#### Effect of molecular sieves (3 Å × 1.5 mm) on the synthesis of ascorbyl palmitate

Varying amounts of molecular sieves (10–100 mg) were added to the reaction mixture. The esterification was carried out using immobilized lipase at 75 °C for 18 h under continuous shaking (120 rpm).

#### Characterization of ascorbyl palmitate

The synthesis of ascorbyl palmitate was done by Fourier transform infrared spectroscopy and the FTIR spectrum was recorded on a Perkin Elmer spectrophotometer in transmittance mode in KBr.

## Results and discussion

### Immobilization of lipase on Celite-545 matrix

The swelling capacity (in 0.05 M Tris buffer; pH 8.5) of Celite 545 in water was recorded as 1.9 times. A commercial lipase “lipolase 100L” (12.02 U/ml) was used for immobilization by adsorption onto the Celite-545 matrix. The matrix treated with 1 % (v/v) glutaraldehyde showed 75 % binding/retention of commercial lipase, while the untreated matrix resulted in 61 % binding/retention of lipase.

### Optimization of the hydrolytic properties of immobilized lipase

#### Effect of pH of reaction buffer on the hydrolytic activity of Celite-bound lipase

**Fig. 2:**
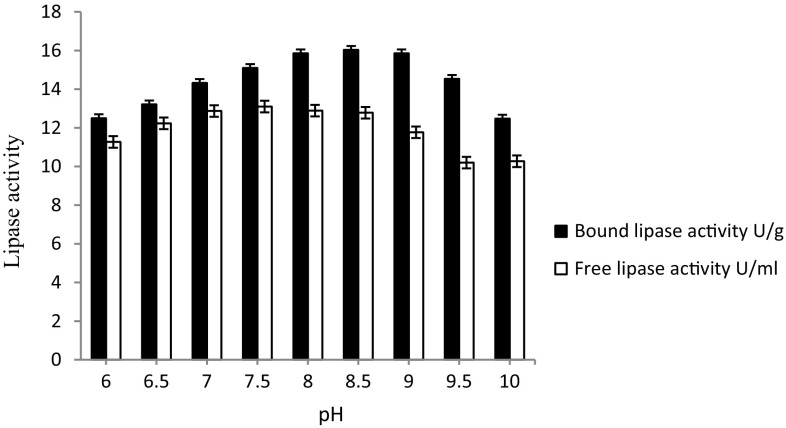
Effect of pH of the reaction buffer on the hydrolytic activity of Celite-bound lipase. The maximum enzyme activity (16.03 ± 0.015 U/g) was recorded at pH 8.5

The maximum enzyme activity (16.03 ± 0.015 U/g) was recorded at pH 8.5. With further change in pH, there was a gradual decrease in enzyme activity. The free enzyme showed maximum activity (13.0 U/ml) at pH 7.5. The Celite-bound lipase showed a shift in the pH of the reaction system toward alkalinity (Fig. 2). In a study (Kumar and Kanwar 2011), NC-membrane bound lipase was more stable at high pH (pH 8.0–9.5), displaying better hydrolytic activity as compared to free lipase which showed less activity at pH 8.5. The lipase immobilized on the mesoporous material of high carbon content showed high activity at pH 7.0 (Claudia et al. 2014).

#### Effect of temperature on the hydrolytic activity of bound lipase

**Fig. 3:**
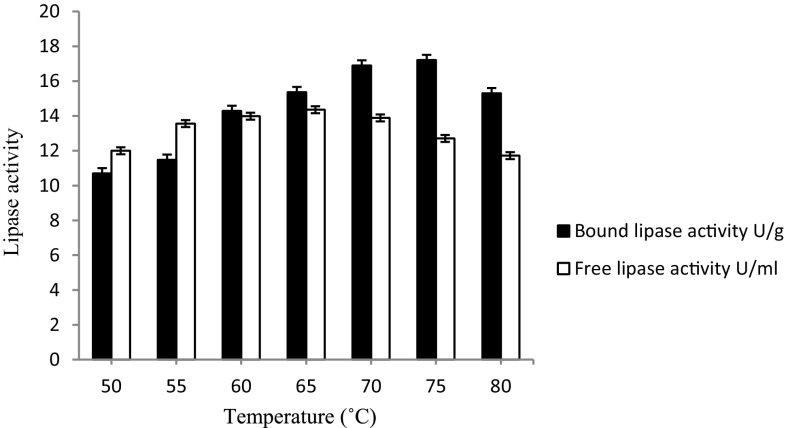
Effect of temperature on the hydrolytic activity of bound lipase. The enzyme activity for Celite-bound lipase showed maximum enzyme activity of 17.2 ± 0.01 U/g at 75 °C

The enzyme activity for Celite-bound lipase showed maximum enzyme activity of 17.2 ± 0.01 U/g at 75 °C (Fig. 3). An increase in the incubation temperature resulted in a gradual decrease in enzyme activity. The free enzyme on the contrary showed maximum activity of 14.36 U/ml at 65 °C and least hydrolytic activity was recorded at 80 °C. Thus, Celite-bound lipase was used for further experiments. The previous study of the lipase from *Bacillus* sp. ITP-001 immobilized in a sol gel matrix showed optimum activity at 80 °C (Carvalho et al. 2013). Temperature also has an important effect on the physical state of substrate dispersion. Higher temperature leads to liquefaction that tends to make substrate more diffusible and easily acceptable to the enzyme. It appeared that the structure of the lipase becomes more fluid and open at an elevated temperature of 70 °C and above that was employed for achieving esterification.

#### Effect of C-chain length (substrate) of *p*-nitrophenyl esters on the hydrolytic activity of immobilized lipase

**Fig. 4:**
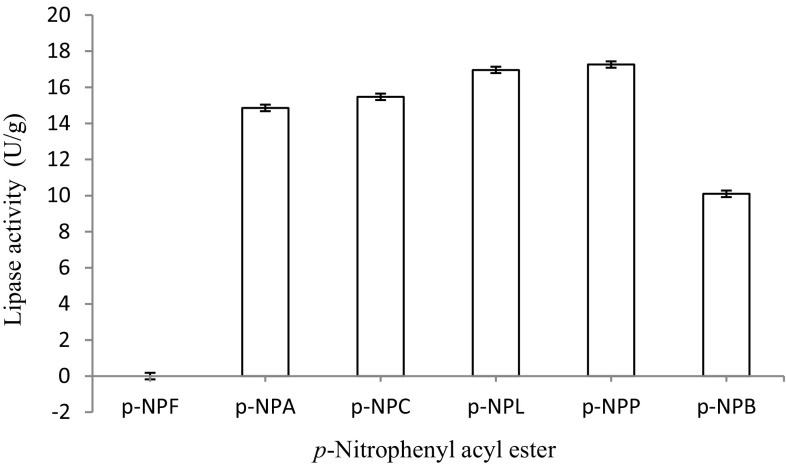
Effect of C-chain length (substrate) of *p*-nitrophenyl esters on the hydrolytic activity of immobilized lipase. The maximum enzyme activity was observed at 75 °C (17.26 ± 0.02 U/g) with *p*-NPP as a substrate

The hydrogel-immobilized lipase showed a variable specificity toward the tested *p*-nitrophenyl esters. The Celite-bound lipase (5 mg) showed a preferential affinity toward esters of relatively longer C-chain length such as *p*-NPP and *p*-NPL. The maximum enzyme activity was observed at 75 °C (17.26 ± 0.02 U/g) with *p*-NPP as a substrate (Fig. 4). The immobilized lipase showed a variable specificity/hydrolytic activity toward various *p*-nitrophenyl esters. The substrate specificity of a lipase is usually determined by the size and the hydrophilicity/hydrophobicity of its pockets. Usually, a tunnel-like binding site was more likely to accept substrates with long-chain fatty acids. It seems that in the present study, among various *p*-nitrophenyl acyl esters, the high C-length (C: 16) ester (*p*-NPP) was more efficiently hydrolyzed than other esters. This indicated a preferential specificity of lipase toward longer carbon chain length substrates. Previously, a lipase from psychrotrophic *Pseudomonas cepacia* immobilized on a commercially available silica support showed a higher activity with *p*-NPA and very low with *p*-NPP (Goncalves et al. 1997).

#### Effect of salt ions on the hydrolytic activity of immobilized lipase

**Fig. 5:**
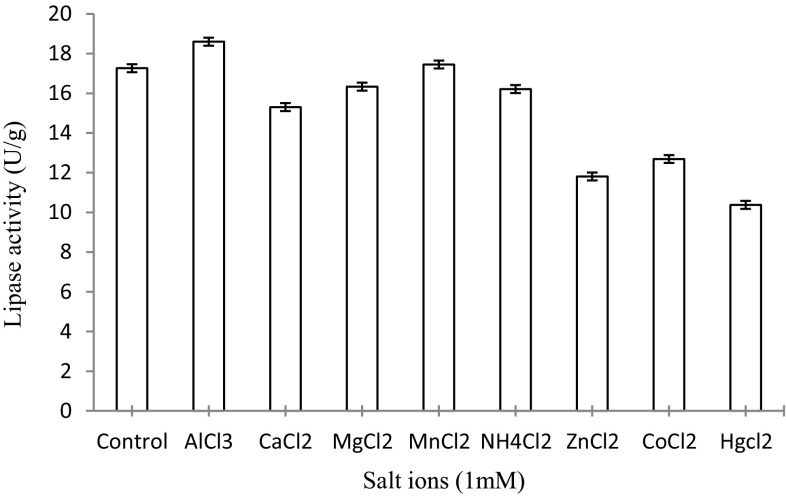
Effect of salt ions on the hydrolytic activity of immobilized lipase. The lipase activity was found to be stimulated only in the presence of Al^+3^ (18.01 ± 0.01 U/g)

The different salt ions often stabilize or destabilize the enzyme structure and hence influence its activity. The results indicated that the lipase was found to be stimulated only in the presence of Al^+3^ (18.01 ± 0.01 U/g) in comparison to the control (17.26 ± 0.01 U/g), whereas other ions inhibited the activity of the lipase (Fig. 5). The presence of metal ions at 1 mM and 5 mM of Mg^2+^ and Ca^2+^ promoted lipase activity from *Bacillus sphaericus* MTCC 7542 (Tamilarasan and Kumar 2011).

#### The effect of chelating agents on the hydrolytic activity of immobilized lipase

The enzyme activity (18.29 ± 0.015 U/g) was slightly increased in the presence of EDTA at 1 mM concentration with respect to the control (17.26 ± 0.01 U/g) (Table 1). However, there was a decrease in the enzyme activity at higher concentration of EDTA, i.e., at 3 mM (16.73 ± 0.02 U/g) and 5 mM (15.83 ± 0.01 U/g). Sodium citrate decreased the enzyme activity at all the tested concentrations, viz. 1 mM (15.97 U/g), 3 mM (14.73 ± 0.02 U/g) and 5 mM (9.83 ± 0.01 U/g) with respect to the control. An increase in the activity of lipase from *Bacillus coagulans* MTCC-6375 has been reported by 20 mM EDTA (Kanwar et al. 2005).

**Table 1:** Effect of chelating agent on bound lipases

Control 17.26 (U/g)	Concentration 1 (mM)	Concentration 3 (mM)	Concentration 5 (mM)
EDTA (U/g)	18.29	16.73	15.83
Sodium citrate (U/g)	15.92	14.73	9.83

#### Effect of detergents on the hydrolytic activity of immobilized lipase

**Fig. 6:**
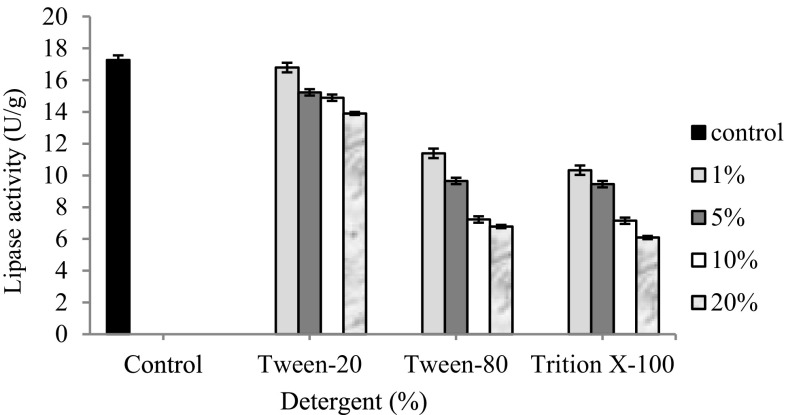
Effect of detergents on the hydrolytic activity of immobilized lipase. All tested detergents reduced the enzyme activity to a considerable extent in comparison to the control (17.26 ± 0.02 U/g)

All the selected detergents (Tween-20, Tween-80 and Triton X-100) had an inhibitory effect on the enzyme activity (Fig. 6). When pre-exposed to Celite -bound lipase (5 mg) at 1, 5, 10 and 20 % (v/v) final concentration (in Tris buffer 0.02 M, 8.5 pH), all tested detergents reduced the enzyme activity to a considerable extent in comparison to the control (17.26 ± 0.02 U/g; Fig. 6) at a temperature of 75 °C. Previously, the presence of Tween 80 caused a sharp decline in the activity of NC-bound lipase (Kumar and Kanwar 2011).

#### Reusability of Celite-immobilized lipase

**Fig. 7:**
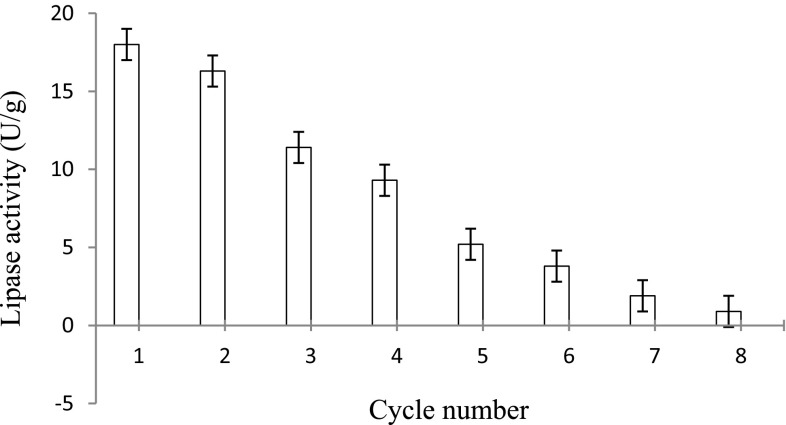
Reusability of Celite-immobilized lipase. The Celite-immobilized lipase retained more than 50 % of its original activity up to the fourth cycle of repetitive reaction and thereafter its activity declined sharply after each cycle of reuse

While studying the effect of reusability under optimized conditions, the recorded lipase activities were 18.0, 16.3, 11.4, 9.3, 5.2, 3.8, 1.9 and 0.9 U/g, respectively, during the first to eighth cycle of reuse of immobilized lipase (5 mg) at 75 °C under shaking (Fig. 7). Thus, it was observed that the Celite-immobilized lipase retained more than 50 % of its original activity up to the fourth cycle of repetitive reaction and thereafter its activity declined sharply after each cycle of reuse. In a previous study, the nitrocellulose-bound lipase was repeatedly used as a biocatalyst to perform hydrolytic reactions. The nitrocellulose-bound lipase retained more than 50 % of its original activity after the fifth repetitive cycle of hydrolysis (Kumar and Kanwar 2011).

### Optimization of esterification conditions for the synthesis of ascorbyl palmitate by the Celite-bound biocatalyst

#### Effect of relative molar concentration of reactants on the synthesis of ascorbyl palmitate

**Fig. 8:**
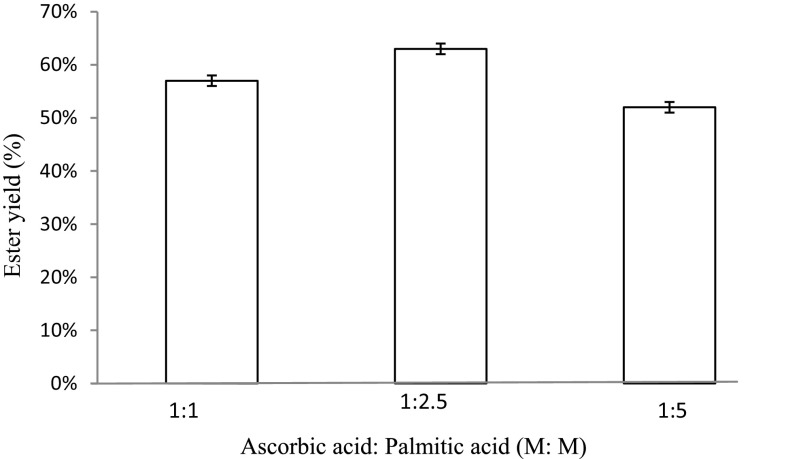
Effect of relative molar concentration of reactants on the synthesis of ascorbyl palmitate. The highest yield of ester was 63 % when ascorbic acid and palmitic acid were used at a concentration of 1 M:2.5 M, respectively, in DMSO

The effect of varying molar concentrations of each of the reactants on ascorbyl palmitate synthesis by Celite-bound lipase was studied by keeping the concentration of one of the reactants (ascorbic acid) constant at 1 M and varying the concentration of the second reactant (1, 2.5 and 5 M). The highest yield of ester was 63 % when ascorbic acid and palmitic acid were used at the concentration of 1 M:2.5 M, respectively, in DMSO (Fig. 8). The esterification was carried out at 75 °C for incubation time of 10 h under continuous shaking using Celite-bound lipase. In another study, a molar ratio of 1:3 (ethanol:propionic acid) was optimum for the synthesis of ethyl propionate in hexane (Kumar et al. 2005). Also, the best reaction conditions for immobilized *Pseudomonas stutzeri* lipase TL were 55 °C, 1:5 ascorbic to palmitic acid molar ratio obtaining 57 % yield of ester (Santibanez et al. 2014).

#### Effect of reaction temperature on the synthesis of ascorbyl palmitate

**Fig. 9:**
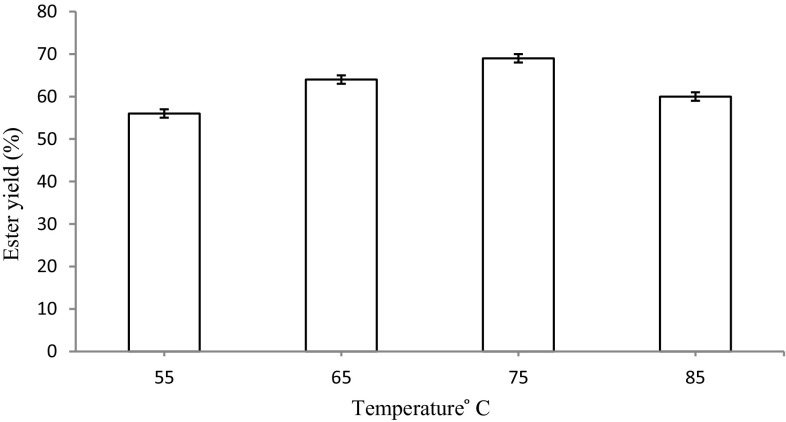
Effect of reaction temperature on the synthesis of ascorbyl palmitate. The maximum yield of ascorbyl palmitate (69 %) was noticed at a temperature of 75 °C

The optimum temperature for the synthesis of ascorbyl palmitate was evaluated by incubating the reaction mixture at 55, 65, 75 and 85 °C in the incubator shaker for 10 h. The maximum yield of ascorbyl palmitate (69 %) was noticed at a temperature of 75 °C. At a temperature higher than 75 °C, a gradual decrease in the ester yield was observed indicating a denaturing effect on the Celite-bound biocatalyst (Fig. 9). Previously, the optimum temperature for butyl ferulate (62 mM) synthesis in DMSO using silica-bound lipase was found to be 45 °C (Chandel et al. 2011).

#### Effect of incubation time for the synthesis of ascorbyl palmitate

**Fig. 10:**
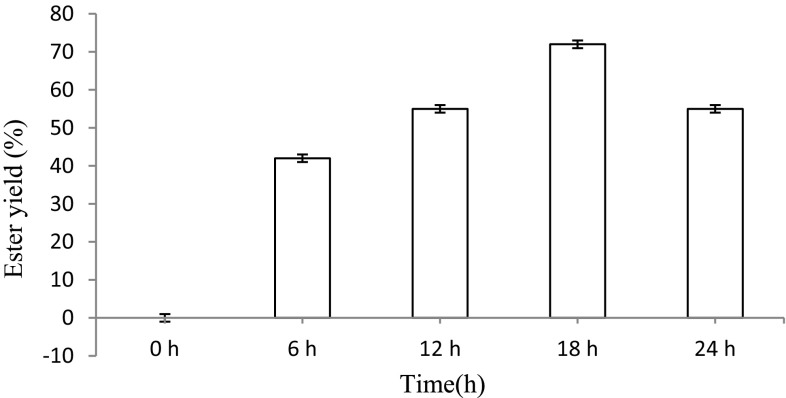
Effect of incubation time for the synthesis of ascorbyl palmitate. The yield of ester was found to be highest after 18 h of reaction resulting in 72 % of ester yield

The optimum reaction time for the synthesis of ascorbyl palmitate by Celite-bound lipase was evaluated by incubating the reaction mixture at 75 °C for different time intervals of 0, 6, 12, 18 and 24 h under continuous shaking (120 rpm) for the synthesis of ascorbyl palmitic acid. The yield of ester was found to be highest after 18 h of reaction, resulting in 72 % of ester yield (Fig. 10). In a recent study, a reaction time of 8 h in case of synthesis of chain of coumarate esters (Sharma et al. 2014) was found.

#### Effect of biocatalyst concentration on the synthesis of ascorbyl palmitate

**Fig. 11:**
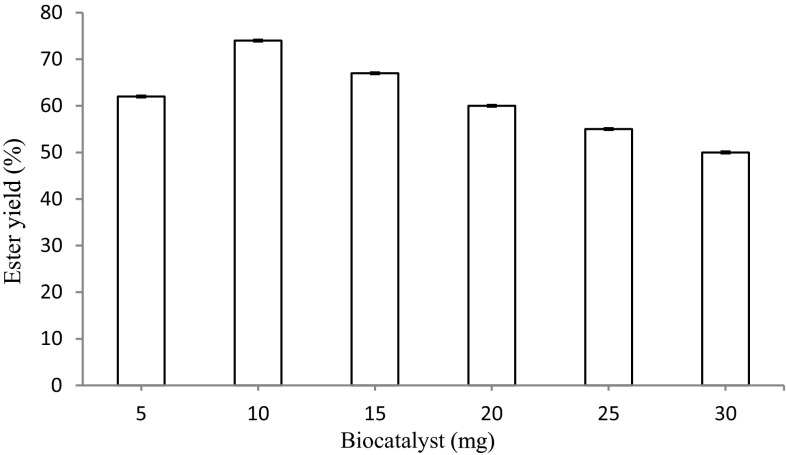
Effect of biocatalyst concentration on the synthesis of ascorbyl palmitate. The maximum ester was formed with 10 mg of enzyme (74 %)

The effect of the amount of biocatalyst used to catalyze the ester synthesis was studied by incubating the reaction mixture with varying concentrations of biocatalyst (5–30 mg) for 18 h at 75 °C. The maximum ester was formed with 10 mg of enzyme (74 %) (Fig. 11) and with any further increase in the amount of biocatalyst a similar amount of ester was produced. Previously, 10 mg/ml (i.e., 1 g/ml) of Celite-bound lipase was found to give an optimum yield of ethyl ferulate in DMSO (Kumar and Kanwar 2010). In a recent study, the production of ascorbyl palmitate from palmitic acid and ascorbic acid through the esterification catalyzed by Novozyme 435 was performed under microwave irradiation; the optimized biocatalyst concentration was 15 % (w/w in relation to ascorbic acid weight) of enzyme at 70 °C and the final 71 % yield of ascorbyl palmitate could be achieved (Ingrid et al. 2014).

#### Effect of molecular sieves (3 Å × 1.5 mm) on the synthesis of ascorbyl palmitate

**Fig. 12:**
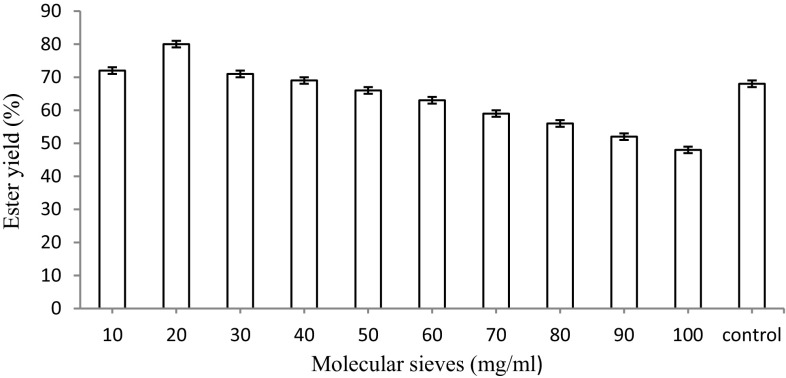
Effect of molecular sieves on the synthesis of ascorbyl palmitate. The maximum increase in the amount of ester yield resulted in 20 mg of molecular sieves (80 %) in comparison to the control (68 %) without molecular sieves

The reaction mixture (2 g) containing 10 mg enzyme, 1 M ascorbic acid and 2.5 M palmitic acid when incubated at 75 °C with varying concentrations of molecular sieves (10–100 mg) resulted in a marked increase in the amount of ester yield with 20 mg of molecular sieves (80 %) in comparison to the control (68 %) without molecular sieves (Fig. 12). Any further increase in the molecular sieves beyond 20 mg caused a gradual to drastic decrease in the amount of ester. An improvement in the rate of esterification has been previously reported for esterification of lauric acid and methanol in the presence of molecular sieves (Mustafa 1998).

#### Characterization of ascorbyl palmitate

**Fig. 13:**
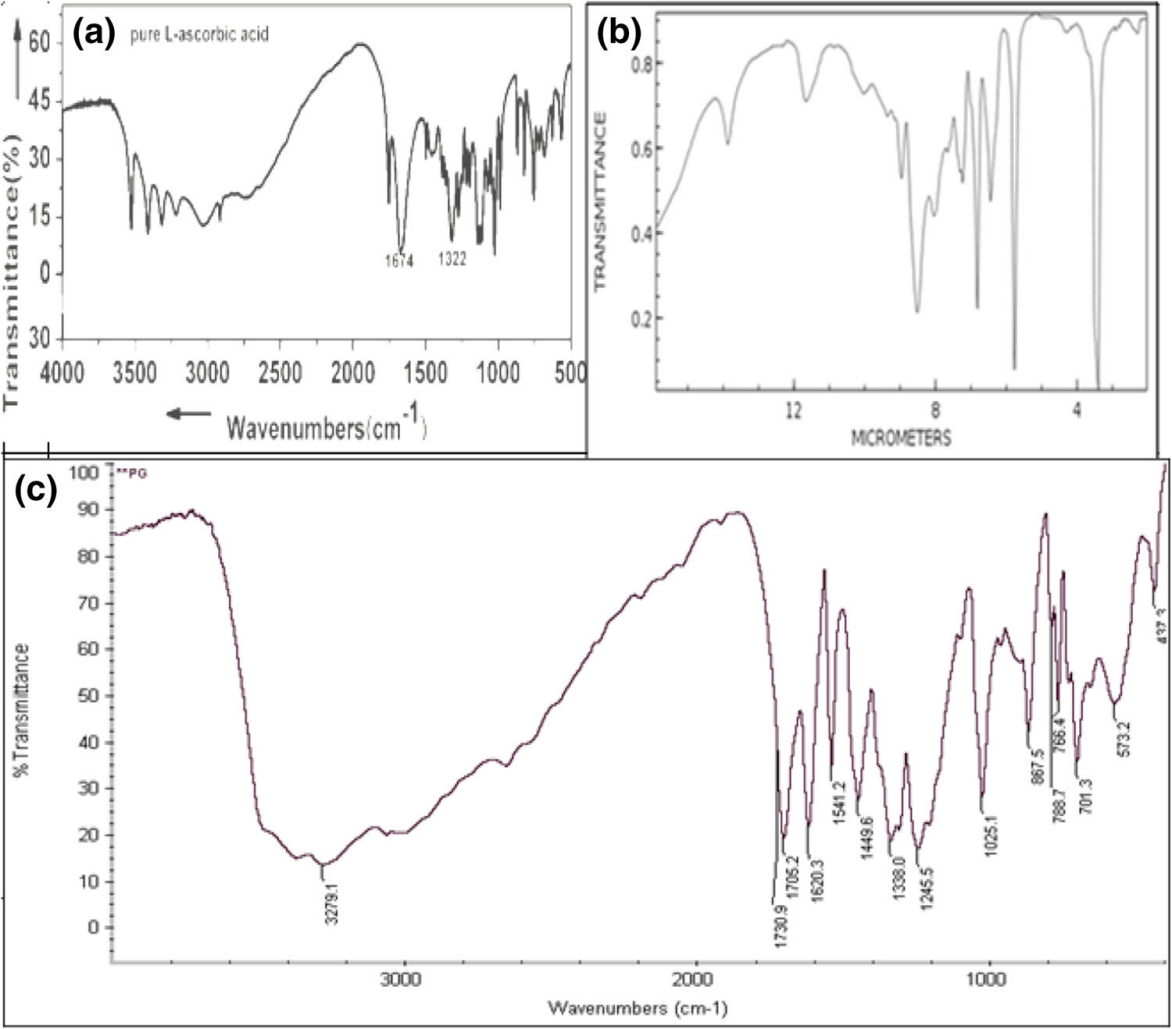
FTIR spectrum: **a** ascorbic acid, **b** palmitic acid, **c** ascorbyl palmitate

FTIR spectrum of ascorbyl palmitate had a sharp peak at 1730.9 cm^−1^ which was ascribed to –C=O stretching of the ester group; when compared with the spectrum of ascorbic acid, this peak of ester was not present and clearly confirmed that the lipase-catalyzed esterification reaction had occurred. The other peak at 2939.5 cm^−1^ was due to the C–H stretching of the methyl group present in ascorbyl palmitate (Fig. 13).

## Conclusion

In the present study, ascorbyl palmitate was synthesized in an organic medium using Celite-bound immobilized commercial lipase (Lipolase 100L). In an aqueous reaction system, the Celite-bound lipase showed maximum activity with *p*-nitrophenyl palmitate (C-16) at an alkaline pH 8.5 and temperature 75 °C. The esterification of ascorbic acid and palmitic acid was successfully achieved with 10 mg/ml immobilized biocatalyst at 75 °C for 18 h under shaking (120 rpm). Molecular sieves had an enhancing effect on the ester synthesis resulting in enhanced yield(s) at a concentration of 20 mg/ml.

## References

[CR1] Carvalho NB, Barbosa JMP, Oliveira MVS, Fricks AT, Soares ALCMF (2013). Biochemical properties of *Bacillus* sp. ITP-001 lipase immobilized with a sol gel process. Quim Nova.

[CR2] Chandel C, Kumar A, Kanwar SS (2011). Enzymatic synthesis of butyl ferulate by silica-immobilized lipase in a non-aqueous medium. J Biomater Nanobiotechnol.

[CR3] Claudia B, Sindy E, Lorena W, Andres I, Monica M (2014). Carbonaceous-siliceous composite materials as immobilization support for lipase from *Alcaligenes* sp.: application to the synthesis of antioxidants. Carbon.

[CR4] Coppen PP, Allen JC, Hamilton RJ (1999). The use of antioxidants, Chapter 5. Rancidity in foods.

[CR5] Garlapati VK, Banerjee R (2013). Solvent-free synthesis of flavour esters through immobilized lipase mediated transesterification. Enzym Res.

[CR6] Goncalves APV, Lopes JM, Lemos F, Ribeiro FR, Prazeres DMF, Chabral JMS, Aires-Barros MR (1997). Effect of the immobilization supports on the hydrolytic activity of a cutinase from *Fusarium solanipisi*. Enzym Microbial Technol.

[CR7] Ingrid CR, Costa ICR, Sutili FK, da Silva GVV, Leite SGF, Miranda LSM, de Souza ROMA (2014). Lipase catalyzed ascorbyl palmitate synthesis under microwave irradiation. J Mol Catal B Enzym.

[CR8] Jacobsen C (2010). Challenges when developing omega-3 enriched foods. Lipids.

[CR9] Kageyama K, Bradbury MJ, Zhao L, Blount AL, Vale WW (1999). Urocortin messenger ribonucleic acid: tissue distribution in the rat and regulation in thymus by lipopolysaccharide and glucocorticoids. Endocrinology.

[CR10] Kanwar SS, Kaushal RK, Jawed A, Chimni SS (2005). Evaluation of methods to inhibit lipase in colorimetric assay employing *p*-nitrophenyl palmitate. Indian J Biochem Biophys.

[CR11] Karmee SK (2011). The synthesis, properties and application of ascorbyl ester. Lipid Technol.

[CR12] Kumar A, Kanwar SS (2010). Synthesis of ethyl ferulate in organic medium using Celite-immobilized lipase. Bioresour Technol.

[CR13] Kumar A, Kanwar SS (2011). Catalytic potential of a nitrocellulose membrane-immobilized lipase in aqueous and organic media. J Appl Polym Sci.

[CR14] Kumar S, Pahujani S, Ola RP, Kanwar SS, Gupta R (2005). Enhanced stability of silica immobilized lipase from *Bacillus coagulans* BTS-3 and synthesis of ethyl propionate. Acta Microbiol Immunol Hung.

[CR15] Liang J, Zeng W, Yao P, Yuanan W (2012). Lipase-catalyzed regioselective synthesis of palmitolyglucose ester in ionic liquids. Adv Biol Chem.

[CR16] Mustafa UU (1998). A study on the lipase catalyzed esterification in organic solvent. Trends J Agric For.

[CR17] Padilha GS, de Barros M, Alegrea RM, Tambourgi EB (2013). Production of ethyl valerate from *Burkholderia cepacia* lipase immobilized in alginate. Chem Eng Trans.

[CR18] Pokorny J, Yanishlieva N, Gordon M (2001). Antioxidants in food: practical applications.

[CR19] Rajesh B, Reddy IB (2013). Lipase from organic solvent tolerant *Bacillus* strain C5: isolation and identification. Int J Sci Res.

[CR20] Santibanez L, Wilson L, Illanes A (2014). Synthesis of ascorbyl palmitate with immobilized lipase from *Pseudomonas stutzeri*. J Am Oil Chem Soc.

[CR21] Sharma S, Kanwar SS (2014). Organic solvent tolerant lipases and applications. Sci World J.

[CR22] Sharma S, Dogra P, Chauhan GS, Kanwar SS (2014). Synthesis of alkyl coumarate esters by lipase of *Bacillus licheniformis* SCD11501. J Mol Catal B Enzym.

[CR23] Stojanovic M, Carevic M, Mihailovic M, Velickovic D, Dimitrijevic A, Milosavic N, Bezbradica D (2015). Influence of fatty acid on lipase-catalyzed synthesis of ascorbyl esters and their free radical scavenging capacity. Appl Biotechnol Biochem.

[CR24] Sun J, Lim Y, Liu SQ (2013). Biosynthesis of flavor esters in coconut cream through coupling fermentation and lipase-catalyzed biocatalysis. Eur J Lipid Sci Technol.

[CR25] Tamilarasan K, Kumar MD (2011). Optimization of medium components and operating conditions for the production of solvent-tolerant lipase by *Bacillus sphaericus* MTCC 7542. Afr J Biotechnol.

[CR26] Wawire M, Oey I, Mathooko F, Njoroge C, Shitanda D, Hendrickx M (2011). Thermal stability of ascorbyl acid and ascorbyl acid oxidase in African cowpea leaves (*Vigna unguiculata*) of different maturities. J Agric Food Chem.

